# Predicting Failure of Active Surveillance in Desmoid-Type Fibromatosis Using Radiomics: An International Multi-center Cohort Study

**DOI:** 10.1245/s10434-026-19931-4

**Published:** 2026-06-11

**Authors:** Stefanie N. Hakkesteegt, Douwe J. Spaanderman, Chiara Colombo, Anne-Rose W. Schut, Andrea Vanzulli, Francesco Barretta, C. Morosi, Marco Fiore, Peter Ferguson, Harini Suraweera, Anthony M. Griffin, Lawrence M. White, Joel Shapiro, Danchen Ge, Dirk J. Grünhagen, Geert J. L. H. van Leenders, David Hanff, Jacob J. Visser, Wiro J. Niessen, Stefan Klein, the Dutch Grafiti Research Group, Anne-Rose W. Schut, Dirk J. Grünhagen, L. Been, M. Bemelmans, J. Bonenkamp, S. Dijkstra, F. van Coevorden, S. N. Hakkesteegt, S. Sleijfer, M. Timbergen, D. van Broekhoven, T. van Dalen, R. van Ginkel, W. van Houdt, J. van der Hage, Cornelis Verhoef, Rebecca A. Gladdy, Alessandro Gronchi, Cornelis Verhoef, Martijn P. A. Starmans

**Affiliations:** 1 Department of Surgical Oncology and Gastrointestinal Surgery, Erasmus MC Cancer Institute, University Medical Center Rotterdam, Rotterdam, The Netherlands; 2 Department of Radiology and Nuclear Medicine, Erasmus MC Cancer Institute, University Medical Center Rotterdam, Rotterdam, The Netherlands; 3 Department of Surgery, Fondazione IRCCS Istituto Nazionale dei Tumori, Milan, Italy; 4 Department of Radiology, Fondazione IRCCS Istituto Nazionale dei Tumori, Milan, Italy; 5 Diagnostic and Interventional Radiology Residency Program, Università degli Studi di Milano, Milan, Italy; 6 Department of Biostatistics for Clinical Research, Fondazione IRCCS Istituto Nazionale dei Tumori, Milan, Italy; 7 Division of Orthopaedic Surgery, Department of Surgery, Sinai Health System, University of Toronto Musculoskeletal Oncology Unit, Toronto, Canada; 8 Department of Surgical Oncology, Department of Surgery, University of Toronto, Toronto, ON Canada; 9 Toronto Joint Department of Medical Imaging, Sinai Health System, Women’s College Hospital,, University Health Network, Toronto, ON Canada; 10 Department of Pathology, Erasmus MC Cancer Institute, University Medical Center Rotterdam, Rotterdam, The Netherlands; 11 Faculty of Medical Sciences, University of Groningen, Groningen, The Netherlands

## Abstract

**Background:**

Active surveillance (AS) is the first-line approach for desmoid-type fibromatosis (DTF). However, 30 % of patients require active treatment. Identifying these patients will help upfront to define a personalized treatment approach. This study assessed whether radiomics can predict AS failure in patients with DTF.

**Methods:**

This multicenter study included data from the Netherlands (NL), Italy (ITA), and Canada (CAN). The study included patients with extra-abdominal DTF initially managed with AS and baseline MRI. Tumors were segmented using a minimally interactive deep-learning method, and radiomics features were extracted from T1-weighted (T1W) and T2-weighted (T2W) MRI scans. Prediction models to predict AS failure versus no failure were created using various machine-learning approaches. Both an internal cross-validation using all available data and an external leave-one-country-out cross-validation were used to assess model performance.

**Results:**

The cohort included 200 patients (72 NL, 62 ITA, 66 CAN), with AS failing for 26 % of the patients. Internal validation of the T1W+T2W imaging model resulted in an overall area under the curve (AUC) of 0.69 (95 % confidence interval [CI] 0.60–0.79). External validation resulted in an AUC of 0.58 (95 % CI 0.42–0.74) in the Dutch cohort, 0.76 (95 % CI 0.60–0.91) in the Italian cohort, and 0.77 (95 % CI 0.65–0.89) in the Canadian cohort. Adding clinical features did not improve the models’ performance.

**Conclusions:**

Predicting AS failure with radiomics showed reasonable performance and generalized well to the Italian and Canadian cohorts. Pending improvements to the model or patient selection, the authors’ model shows potential to better identify which DTF patients will benefit from AS and which will not.

**Supplementary Information:**

The online version contains supplementary material available at 10.1245/s10434-026-19931-4.

Desmoid-type fibromatosis (DTF) is a rare, locally aggressive soft-tissue tumor classified as a borderline neoplasm.^1^ Despite its lack of metastatic potential, DTF can display locally infiltrative growth and is characterized by a high local recurrence risk, particularly after surgical intervention.^2^ The clinical course is highly variable and unpredictable, with tumors exhibiting phases of initial progression, growth stabilization, or spontaneous regression without any form of therapy.^3–5^ This variation in tumor behavior and clinical presentation poses a major challenge in DTF management.

Currently, international guidelines recommend active surveillance (AS) as the preferred initial management strategy for asymptomatic or mildly symptomatic patients.^6^ During AS, patients are typically monitored at regular intervals for several years, depending on symptom progression and tumor behavior. Approximately 30 % of patients ultimately switch to active treatment consisting of systemic treatment, surgery, or other local therapies due to radiologic and/or clinical progression.^3,7^ At the same time, 70 % of patients never require active treatment and are consequently subjected to unnecessary imaging and outpatient visits.

A main clinical need is to identify patients likely to do well with AS, thereby reducing the burden of prolonged follow-up evaluation and optimizing health care resources. In this context, a predictive model could support patient counseling by providing an objective rationale for early discharge, reinforcing individualized care.

Several clinicopathologic predictive factors for the initiation of active treatment, defined in this study as failure of AS, have been reported, including tumor diameter, anatomic location, and CTNNB1 mutation status^7^. Furthermore, MRI features have shown potential for predicting tumor behavior in DTF during AS. Among these features, T2 signal intensity is an important predictor of tumor activity, with hyperintense regions corresponding to active cellular components and low-intensity areas reflecting fibrous, inactive tissue.^8,9^ Post-contrast enhancement similarly highlights active tumor regions.^10^ Assessing these imaging characteristics quantitatively through radiomic analysis may provide a more precise evaluation of tumor behavior and support decision-making during AS.

Radiomics is a rapidly evolving field that extracts quantitative features from medical imaging. It is based on the hypothesis that imaging features reflect underlying tumor biology, including genetic mutations and cellular architecture.^11^ In the context of DTF, radiomics research remains limited primarily due to the rarity of the disease and limited data.^12^ Nevertheless, radiomic analyses have shown promise in recent DTF studies, outperforming standard Response Evaluation Criteria in Solid Tumors (RECIST) criteria for tumor progression prediction, differentiating DTF from soft tissue sarcoma and demonstrating potential for predicting tumor behavior.^13–15^ However, these findings have not undergone external validation to date. Moreover, although these results indicate that MRI-derived radiomic features may have diagnostic and prognostic value in DTF, it remains unknown whether radiomics can accurately predict disease deterioration and guide decisions on which patients require continued follow-up evaluation versus those eligible for early discharge.

The primary aim of this study was to develop and externally validate a radiomics model to predict which patients are eligible for early discharge from follow-up evaluation due to a low risk of AS failure. The secondary aim was to develop and externally validate a radiomics model to predict tumor progression.

## Methods

**Fig. 1:**
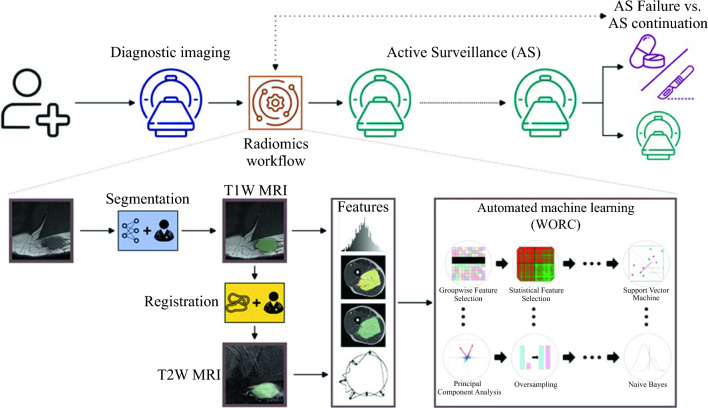
Radiomics workflow to predict active surveillance (AS) failure in desmoid-type fibromatosis (DTF). Patients undergo baseline MRI, and tumors are segmented using a minimally interactive deep-learning tool, propagated to other sequences via rigid registration, and quality-checked. From these, 1128 radiomics features (intensity, shape, texture) are extracted and processed with the Workflow for Optimal Radiomics Classification (WORC) toolbox, which selects features, resamples data, and evaluates 1000 machine-learning pipelines to build an ensemble model predicting AS outcome^25^

A schematic overview of the methodology is depicted in Fig. 1.

### Study Samples

Data from high-volume sarcoma centers in the Netherlands, Italy and Canada were used to form three study cohorts. Failure of AS was defined as the initiation of any type of active treatment (i.e., systemic therapy, surgery, or other local treatment) after initial management with AS due to symptomatic progression or radiologic progressive disease according to RECIST, in agreement with international guidelines.

The study included patients with a histologically confirmed diagnosis of extra-abdominal DTF who were initially managed with AS and had a baseline MRI. If no baseline MRI was available, the first follow-up MRI was used, provided it was performed within 1 year after diagnosis and before AS failure. For the tumor progression prediction analysis, patients were required to have undergone at least 1 year of AS, unless progression occurred earlier. The absence of both T1w and T2W sequences, poor-quality scans, and incomplete depiction of the tumor were exclusion criteria.

The first cohort was created with data from the Netherlands on patients previously included in a prospective observational trial in the period 2014–2018,^3^ which assessed the feasibility of AS for extra-abdominal DTF. The second cohort contained patients previously included in a similar prospective observational trial conducted in Italy in the period 2013–2018.^4^ The third cohort was formed with patients who received diagnosis and treatment at the Princess Margaret Cancer Centre and Mount Sinai Hospital, Toronto, Canada. For this cohort, data were extracted from a database that had been prospectively maintained since 2009 according to the same inclusion and exclusion criteria.

Collected clinical variables included age at diagnosis, sex, tumor location, mutation status, tumor size (the largest dimension) on first imaging, tumor behavior during the follow-up period, time between first imaging and first tumor progression, treatment characteristics during follow-up evaluation, and the interval between first imaging and initiation of active treatment.

Tumor behavior was assessed according to RECIST version 1.1^16^ and classified as progressive disease (PD), stable disease (SD), partial response (PR), or complete response (CR). These were further grouped as PD or no PD (comprising SD, PR, and CR). Tumor dimension measurements were obtained from routine clinical radiology reports at each participating institution, after which longitudinal measurements were reviewed by the study team to determine RECIST-defined response.

The study collected MRI scans obtained closest to the time of diagnosis and categorized MRI sequences as T1w (T1), T1 with fat saturation (T1-FS), T2w (T2), or T2 with fat saturation (T2-FS). For analysis, T1 and T1-FS were combined into a T1 model, and T2 and T2-FS were combined into a T2 model.

De-identified imaging data were transferred between institutions using Health-RI XNAT (Extensible Neuroimaging Archive Toolkit).^17,18^ The study team reviewed MR examinations both before image transfer, to confirm availability of required sequences, and after transfer, to assess image quality and suitability for analysis.

Ethical approval was obtained at the participating centers (MEC-2014-124, NCT 02547831, UHN REB 24-5129, MSH REB 24-0065-C) in accordance with local institutional requirements. For all the cohorts, the requirement for individual informed consent for this study, including secondary use and international transfer of de-identified imaging data, was waived by the respective research ethics boards. The study was conducted in accordance with the Declaration of Helsinki, and no identifiable patient information was shared.

### Segmentation

Tumor segmentation was performed using InteractiveNet, a previously developed, minimally interactive deep learning-based tool trained specifically for soft tissue tumors including desmoid-type fibromatosis.^19^ The method requires only six user-defined points placed at the tumor’s extreme boundaries, two in each direction. Segmentations were generated by a clinician (PhD candidate with an MD degree) on the MRI sequence in which the tumor was most clearly visible.

To propagate the segmentations to all other available MRI sequences, rigid transformations were computed using automated image registration software (Elastix^20^) with default settings, aligning each sequence to the segmented sequence in the same imaging plane. The registered segmentations were subsequently reviewed for quality and accuracy.

### Radiomics

The Workflow for Optimal Radiomics Classification (WORC) framework was used to automatically construct and optimize the radiomics in Python (version 3.11).^21^ For each lesion, the default set of 564 features was extracted using PyRadiomics and PREDICT capturing intensity, shape, and texture characteristics (Supplementary Material 1).

As described by Starmans et al.,^22^ manually designing radiomics models through heuristic trial and error is time-consuming, non-reproducible, and prone to overfitting and does not guarantee optimal performance.

To address these limitations, we used WORC to automate and optimize the entire model development process.^21^ The WORC framework standardizes key radiomics components, including feature selection, resampling, and classification, and explores a wide range of commonly used algorithms and hyperparameters for each step. Leveraging automated machine-learning, WORC evaluates 1000 randomly sampled radiomics workflows, each representing a unique combination of algorithms and hyperparameters. The final model is an ensemble of the top 100 performing workflows, combining their posterior probabilities to improve robustness. All code for feature extraction and model construction is available open-source.^23^

### Experimental Setup

For all experiments, a leave-one-country-out cross-validation was used. In each fold, radiomics workflows were trained and optimized using data from two centers to classify AS failure, with the third center serving as an independent external validation set. This procedure was repeated three times such that each center was used once as an independent external validation set, ensuring a robust evaluation of generalizability across institutions.

In all evaluation setups, several radiomics models were constructed using different input data combinations. First, two single-sequence models were evaluated using either T1w or T2w MRI alone. Second, a multi-sequence model combining both T1w and T2w was assessed. If a sequence was missing for a patient, the corresponding feature values were automatically imputed using several imputation strategies, as described by Starmans et al.^21^ Third, a clinical feature model, based on age, sex, tumor location, and CTNNB1, was developed to compare the predictive value of clinical data relative to imaging features. Finally, a combined model integrating both clinical and imaging features was evaluated to explore potential performance improvements from multimodal input.

To assess the impact of external validation, additional experiments were conducted for the single- and multi-sequence models using internal cross-validation on the full dataset pooled across all centers. Comparison between the internal and external validation performance can reveal potential generalization gaps. Finally, the robustness of the radiomics models was evaluated across various patient subgroups (Supplementary Materials 1).

### Evaluation and Statistical Analysis

Model performance in the independent external validation setting was estimated using stratified bootstrap resampling (1000 iterations). For the experiments in which all data were pooled, the corrected resampled *t* test was applied based on 100 random-split cross-validation iterations. Both procedures are part of the default evaluation in WORC^21^.

The performance of the radiomics models was assessed using several metrics, including the area under the receiver operating characteristic (ROC) area under the curve (AUC), sensitivity, specificity, and balanced classification accuracy (BCA). For each model, performance across the external datasets or mean performance across cross-validation folds was reported. To quantify the precision of these estimates, 95 % confidence intervals were computed. Radiomics model calibration was assessed using calibration curves and quantified with the Brier score. Clinical and demographic characteristics of the cohort were analyzed using descriptive statistics in R (version 4.3.1). Categorical variables were reported as absolute numbers and percentages and compared across groups using the standardized mean difference and the chi-square test. Continuous variables were summarized as medians and interquartile ranges (IQR) and compared using the Kruskal–Wallis test due to non-normal distribution.

Imaging features were subjected to univariate statistical analysis to evaluate their association with AS failure. Mann–Whitney *U* tests were applied for each feature, and the resulting *P* values were corrected for multiple comparisons using the Bonferroni correction.

Within each external validation set, the performance (AUC) of the combined T1w+T2w radiomics model was statistically compared with that of other models (e.g., the clinical model based on age, sex, and tumor location) using the DeLong test. All *P* values lower than 0.05 were considered statistically significant.

## Results

### Characteristics of the Study Cohort

**Fig. 2:**
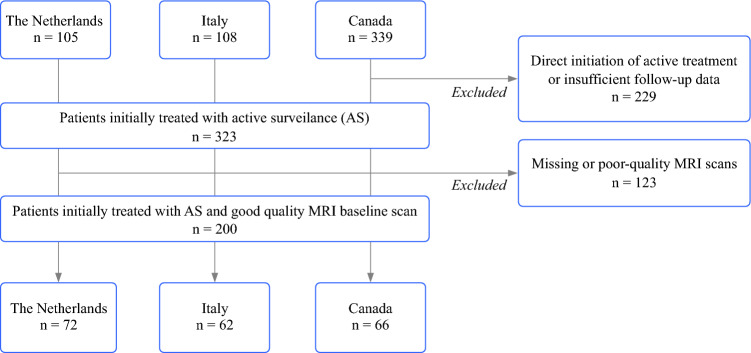
Flowchart depicting the selection of the total study cohort and the three separate validation cohorts, including data from the Netherlands, Italy, and Canada

The two previously conducted prospective observational studies from the Netherlands and Italy included 105 and 108 patients, respectively. These cohorts were screened for the availability of a baseline or first follow-up MRI scan. In addition, 339 patients from the Canadian institutional database were screened for eligibility according to the predefined criteria. Among these patients, 229 were excluded due to immediate initiation of active treatment or insufficient follow-up evaluation. An additional 123 patients were excluded from the combined cohorts due to missing or poor-quality MRI scans. The final study population consisted of 200 patients: 72 from the Netherlands, 62 from Italy, and 66 from Canada (Fig. 2).

Statistically significant differences were found among the cohorts in tumor location and time from diagnosis to imaging (Table 1). Furthermore, mutation status was assessed only in the Dutch and Italian cohorts, in line with the trial designs, and was not available for the Canadian cohort because mutation status testing was not part of standard care. The three cohorts showed substantial heterogeneity in MRI acquisition parameters, including scanner manufacturer, magnetic field strength, sequence availability, and imaging settings (all *P* < 0.01; Table S1).

**Table 1:** Patient characteristics of the three study cohorts: the Netherlands, Italy and Canada^a^

	The Netherlands^b^ (*n* = 72) *n* (%)	Italy^b^ (*n* = 62) *n* (%)	Canada^b^ (*n* = 66) *n* (%)	Total cohort^b^ (*n* = 200) *n* (%)	SMD	*P* Value^c^
Sex ratio (M:F)	14:58	9:53	16:50	39:161	0.147	0.469
Median age at diagnosis: years (IQR)	36.0 (32.0–46.2)	39.0 (34.0–46.0)	38.5 (32.2–47.0)	38.0 (33.0–47.0)	0.127	0.410
Tumor location					0.593	**0.006**
Abdominal wall	28 (39)	35 (57)	47 (71)	110 (55)		
Extremities	19 (26)	9 (14)	7 (11)	35 (17)		
Chest wall	15 (21)	16 (26)	7 (11)	38 (19)		
Breast	5 (7)	0	2 (3)	7 (4)		
Head/neck	5 (7)	2 (3)	3 (4)	10 (5)		
CTNNB1 mutation status					0.454^d^	0.390^e^
T41A	38 (53)	32 (52)	0	70 (35)		
S45F	8 (11)	6 (10)	0	14 (7)		
S45P	12 (17)	12 (19)	0	24 (12)		
WT	6 (8)	7 (11)	0	13 (6)		
Other	3 (4)	5 (8)	0	8 (4)		
Unknown	5 (7)	0	66 (100)	71 (36)		
Median tumor size at intake: cm (IQR)^f^	4.5 (3.2–7.2)	5.2 (3.7–8.0)	6.0 (4.5–7.5)	5.5 (3.6–7.7)	0.127	0.172
Median follow-up period: months (IQR)	33.0 (15.8–44.2)	39.0 (17.0–55.0)	34.0 (24.0–59.0)	35.0 (19.0–51.0)	0.423	**0.020**
Median time between diagnosis and first available imaging: months (IQR)	4.1 (1.1–6.5)	1.4 (0.8–2.0)	2.5 (1.1–4.4)	1.9 (0.8–4.5)	0.488	**<0.001**
AS failure during follow-up	20 (28)	14 (23)	18 (27)	52 (26)	0.169	0.621
Median time to AS failure: months (IQR)	9.5 (5.8–14.2)	8.5 (5.2–15.8)	14.0 (9.0–21.2)	10.0 (6.0–16.0)	0.402	0.181
RECIST PD during follow-up	27 (38)	25 (40)	22 (33)	74 (37)	0.097	0.711
Median time to first RECIST progression: months (IQR)	7.0 (4.5–13.5)	5.0 (4.0–10.0)	6.5 (4.0–10.8)	7.0 (4.0–11.0)	0.100	0.442

SMD, standardized mean difference; IQR, interquartile range; AS, active surveillance; RECIST, Response Evaluation Criteria in Solid Tumors; PD, progressive disease

^a^Ethnicity data were not routinely collected for the study cohorts.

^b^For statistical testing, the Chi-squared and Kruskal–Wallis test were used unless otherwise indicated.

^c^*P* Values lower than 0.05 are presented in bold.

^d^The Canadian cohort was excluded from the SMD analysis.

^e^Fisher’s exact test between the Dutch and Italian cohort. The Canadian cohort was excluded because gene mutation analysis was not performed.

^f^The largest 2D dimension measured on first available MRI used for radiomics analysis

### Radiomics Model Development and Validation

**Fig. 3:**
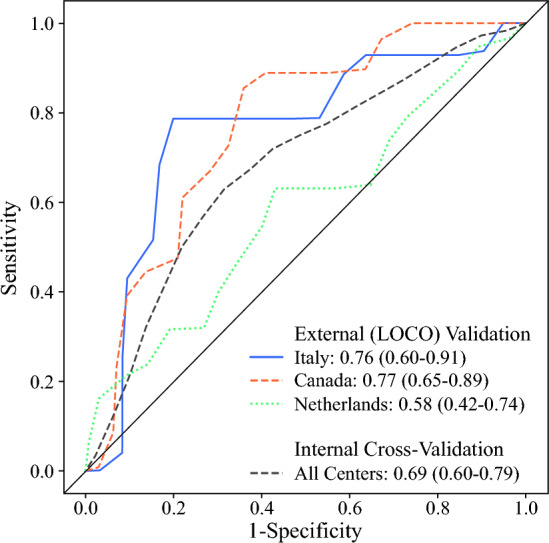
Receiver operating characteristic (ROC) curves of the radiomics model classifying failure versus no failure of active surveillance (AS) based on T1- and T2-weighted imaging features.**Model performance is reported as the area under the ROC curve (AUC) with corresponding 95 % confidence intervals (CI). For the external leave-one-country-out (LOCO) validation across different cohorts, 95 % confidence intervals [CIs] were calculated using ×1000 bootstrapping. For the internal cross-validation experiment, in which all cohort data were combined, 95 % CIs were derived from ×100 repeated cross-validation test folds, ensuring that the model had no prior exposure to the images used for prediction. LOCO, leave-one-country-out

Performance metrics for all the models are summarized in Table 2, and ROC curves are shown in Fig. 3. In the leave-one-country-out validation using features derived from combined T1w and T2w sequences, prediction of AS failure was good for the Italian (AUC, 0.76; 95 % CI 0.60–0.91) and Canadian (AUC, 0.77; 95 % CI 0.65–0.89) cohorts, but was poor for the Dutch cohort (AUC, 0.58; 95 % CI 0.42–0.74). Models based solely on clinical variables performed poorly for the Italian (AUC, 0.64; 95 % CI 0.44–0.83) and Canadian (AUC, 0.66; 95 % CI 0.51–0.82) cohorts. Interestingly, clinical features outperformed the imaging model for the Dutch cohort (AUC, 0.74; 95 % CI 0.59–0.88).

**Table 2:** Performance metrics of the radiomics models, including clinical, single, and multi-sequence models predicting failure of active surveillance (AS) for the three separate cohorts (external validation)

Cohort	AUC^a^	BCA^a^	Sensitivity^a^	Specificity^a^	*P* Value^b^
*Cohort 1: The Netherlands*					
T1 and T2 imaging model	0.58 (0.42–0.74)	0.56 (0.44–0.68)	0.32 (0.10–0.53)	0.80 (0.69–0.91)	
Clinical model	0.74 (0.59–0.88)	0.67 (0.55–0.79)	0.42 (0.19–0.65)	0.92 (0.85–0.99)	0.14
Imaging and clinical model	0.59 (0.43–0.74)	0.53 (0.42–0.65)	0.26 (0.06–0.46)	0.80 (0.69–0.91)	0.71
T1 imaging model^c^	0.56 (0.40–0.73)	0.50 (0.41–0.59)	0.12 (0.00–0.27)	0.89 (0.80–0.98)	
T2 imaging model^c^	0.62 (0.41–0.82)	0.68 (0.51–0.84)	0.55 (0.24–0.85)	0.81 (0.68–0.94)	
*Cohort 2: Italy*					
T1 and T2 imaging model	0.76 (0.60–0.91)	0.66 (0.52–0.80)	0.43 (0.16–0.70)	0.90 (0.81–0.98)	
Clinical model	0.64 (0.44–0.83)	0.57 (0.45–0.68)	0.21 (0.01–0.43)	0.92 (0.84–1.00)	0.06
Imaging and clinical model	0.76 (0.59–0.94)	0.71 (0.57–0.85)	0.50 (0.23–0.77)	0.92 (0.84–0.99)	0.31
T1 imaging model^c^	0.77 (0.59–0.96)	0.57 (0.45–0.70)	0.18 (0.00–0.42)	0.97 (0.90–1.00)	
T2 imaging model^c^	0.78 (0.62–0.94)	0.73 (0.59–0.87)	0.57 (0.31–0.84)	0.89 (0.80–0.98)	
*Cohort 3: Canada*					
T1 and T2 imaging model	0.77 (0.65–0.89)	0.51 (0.45–0.57)	0.06 (0.00–0.16)	0.96 (0.90–1.00)	
Clinical model^d^	0.66 (0.51–0.82)	0.53 (0.42–0.64)	0.22 (0.02–0.42)	0.83 (0.73–0.94)	0.28
Imaging and clinical model^c^	0.77 (0.66–0.88)	0.52 (0.44–0.61)	0.11 (0.00–0.26)	0.94 (0.87–1.00)	0.91
T1 imaging model^c^	0.74 (0.60–0.88)	0.61 (0.47–0.74)	0.44 (0.19–0.69)	0.77 (0.65–0.90)	
T2 imaging model^c^	0.73 (0.58–0.88)	0.55 (0.46–0.65)	0.13 (0.00–0.31)	0.98 (0.93–1.00)	

AUC, area under the receiver operating characteristic (ROC) curve; BCA, balanced classification accuracy.

^a^Data are reported as mean (95 % confidence intervals) over bootstrap resampling iterations. The clinical model contained age, sex, and tumor location features.

^b^The reported *P* values represent DeLong’s test comparing each model against the T1+T2 imaging model.

^c^Sample sizes vary slightly between T1-, T2-, and multi-sequence (T1+T2) models due to missing sequences. Missing features were imputed for the multi-sequence model, enabling inclusion of all patients. These differences prevented DeLong test comparisons.

^d^Clinical model without CTNBB1 mutation status

Incorporating clinical features into combined clinical-radiomics models resulted in no performance gains (Netherlands [0.59; 95 % CI 0.43–0.74], Italy [0.76; 95 % CI 0.59–0.94], Canada [0.77; 95 % CI 0.66–0.88]). Cross-validation on the pooled dataset yielded performance comparable with external validation (Netherlands [0.56; 95 % CI 0.43–0.67], Italy [0.73; 95 % CI 0.61–0.89], Canada [0.74; 95 % CI 0.65–0.84]), suggesting good generalizability because no performance drop was observed in external validation.

The ROC curves for models based on single-sequence features, presented in Fig. S1, showed no substantial difference compared with the multi-sequence radiomics model. The multi-sequence model demonstrated good calibration, with a Brier score of 0.18 (Fig. S2).

Feature importance analysis identified a number of potentially relevant features, but none remained significant after Bonferroni correction (Fig. S3). Notably, the Italian and Canadian cohorts shared several overlapping significant features, whereas the Dutch data showed only a limited number of relevant features.

The prediction of PD was unsuccessful, with poor model performance across all centers. None of the imaging models, clinical models, or combined models achieved meaningful predictive performance. Detailed performance metrics, including center-specific AUCs, are presented in Table S2.

Subgroup analysis showed a similar performance in most subgroups, with most confidence intervals overlapping and none of the models performing substantially worse than the overall model, thus suggesting no biases (Supplementary Materials 1).

## Discussion

The objective of this study was to develop and externally validate MRI-based radiomics models to predict AS failure in DTF patients. Our models demonstrated a promising performance in predicting AS failure in two out of the three centers included. Our study is therefore the first to show the potential of radiomics for predicting AS outcomes, motivating further research into improvement of overall performance and generalizability.

The clinical utility of the developed radiomics model lies primarily in identifying patients with a high probability of AS success because these can be considered suitable candidates for early discharge from routine follow-up evaluation. Currently, all patients are monitored at frequent intervals for several years to assess tumor growth and symptom deterioration. Given that approximately 30 % of patients eventually undergo active treatment after initial surveillance,^3,7^ the remaining patients are subjected to unnecessary follow-up appointments and imaging procedures. Reducing these unnecessary interventions not only would lower health care costs and reduce the burden on clinical resources, but also would lessen patient anxiety because each appointment, each imaging session, and generally being in active surveillance can be a source of significant stress.^24^ In this context, our model can potentially be used to counsel patients by providing an additional, objective argument in favor of discharge, supporting personalized care. Especially patients who are anxious about the course of their disease and feel insecure about discontinuation of their follow-up evaluation may benefit when our model is used in conjunction with the treating physician’s clinical expertise and personalized guidance regarding when to reinitiate contact with the clinic.

On the other hand, the model also holds clinical value in identifying patients likely to experience AS failure. Early identification of this subgroup could support closer monitoring and timely discussion of potential treatment options, allowing for prompt initiation of active treatment once symptoms develop or AS is no longer feasible. In this context, the model may help optimize patient counseling and reduce the psychological burden associated with uncertainty while ensuring that treatment decisions remain symptom-driven and aligned with current management principles.

The imaging models showed reasonable performances and outperformed the clinical model, suggesting that imaging contains more predictive information than can be obtained from clinical variables or CTNNB1 mutation status alone in the Canadian and Italian cohorts. In contrast, for the Dutch cohort, incorporating imaging features reduced the performance compared with the clinical-only model. Interestingly, both single-sequence (T1W or T2W) models performed similarly to the combined T1W- and T2W-imaging model. This was unexpected because previous studies reported that T2W hyperintense signal correlates with more cellular, biologically active tumor components in terms of RECIST progression in DTF patients.^8,9^ Given this association, it would be reasonable to expect that the T2W-imaging model might be better suited to predict AS failure, which is often caused by progressive or symptomatic disease.

Additionally, our radiomics model predicting PD failed to perform well, in line with previous work in which no independent clinical predictors for PD could be identified.^7^ Together, these findings indicate that both the T1W and T2W sequences hold valuable information for medical image analysis in this patient population. Advanced sequences such as dynamic contrast-enhanced MRI and diffusion-weighted imaging can provide additional detailed information on tumor behavior, and incorporating these sequences could potentially improve the model’s performance.^25,26^

The substantially lower model performance observed in the Dutch cohort relative to the Italian and Canadian cohorts may be explained by several factors. Whereas the Italian and Canadian cohorts shared several overlapping significant features, the Dutch data lacked this consistency, possibly reflecting underlying differences in patient or tumor characteristics. The Dutch dataset included a higher proportion of chest wall and “other” locations (including breast and head/neck). In addition, the proportion of abdominal wall tumors, generally associated with more favorable outcomes, differed substantially across cohorts, which may have influenced both baseline outcome distributions and apparent model performance.

Subgroup analyses (Supplementary Materials 1) showed substantial variation by anatomic site, with extremities (AUC, 0.77) and abdominal wall (AUC, 0.72) showing good performance and with chest wall (0.61) and other locations (0.41) performing poorly. Although this raises the possibility that performance differences between cohorts partly reflect anatomic distribution rather than purely imaging-based discrimination, comparison with a clinical model including anatomic location and the leave-one-country-out validation across markedly different site distributions suggests that the radiomics model captures additional tumor-specific imaging information beyond location alone. Additionally, technical imaging parameters influenced performance, with higher field strength (>1.5T [AUC, 0.83] vs ≤1.5T [AUC, 0.65]) associated with better discrimination.

These findings should be interpreted in light of the relatively small and heterogeneous cohorts with variability in imaging protocols and clinical presentation. The lower performance in certain anatomic sites may reflect limited representation and acquisition differences rather than intrinsic limitations of radiomics. Importantly, despite this heterogeneity across three independent cohorts, radiomics demonstrated discriminatory value, highlighting its potential with improved standardization and larger, more balanced datasets. Furthermore, the Dutch cohort had the largest interval between diagnosis and first imaging, which could have led to certain radiomics features linked to progression being lost in time. In addition, the Dutch cohort combined data from seven different hospitals in the Netherlands. Therefore, heterogeneity in MRI acquisition protocols such as differences in scanner vendor, field strength, and sequence parameters may have introduced imaging variability that reduced model consistency. Both the Italian and Canadian cohorts contained data from two different hospitals. Together, these differences likely contributed to the reduced predictive performance in the Dutch cohort.

Several limitations of this study must be acknowledged. First, there was a large imbalance between cases in which AS failed and cases in which it was successful. Although machine-learning techniques were used to mitigate this, the models still may perform better in identifying successful AS cases. This is also reflected in the ROC curves, which primarily capture discrimination and may mask reduced sensitivity in detecting AS failure. Second, mutation status was available only for the Dutch and Italian cohorts due to the design of the original prospective trials and was unavailable for the Canadian cohort. Third, the Canadian cohort was retrospective and differed in study design from the prospective Dutch and Italian cohorts. Nevertheless, uniform inclusion and exclusion criteria were applied across all cohorts, thereby minimizing heterogeneity and likely limiting any impact on the results. Fourth, the radiomics analysis was based on a single MRI time point, thereby limiting the potential to capture dynamic tumor behavior. However, working with a single time point reflects clinical practice, in which the clinician ideally assesses the disease course based on one scan during the first consultation.

In conclusion, this study was the first to evaluate radiomics-based prediction of AS failure in DTF in a large, international, multicenter cohort. Whereas predicting AS failure using baseline MRI scans appeared challenging in the Dutch data, this appeared more feasible in the Italian and Canadian cohorts, offering potential clinical value in identifying low-risk patients who may be eligible for early discharge from follow-up evaluation. Particularly patients who are anxious about the course of their disease or feel uncertain about hospital discharge may benefit from such an objective tool, especially when combined with the treating physician’s clinical expertise. Further refinement and improvement of the model are warranted before consideration of clinical implementation.

## Electronic supplementary material

Below is the link to the electronic supplementary material.

Supplementary Material
