# Supplementary material for: Predicting Failure of Active Surveillance in Desmoid-Type Fibromatosis Using Radiomics: An International Multi-center Cohort Study
Source: Ann Surg Oncol. 2026 Jun 11;33(9):8591–600. doi: 10.1245/s10434-026-19931-4 (PMC13452830; doi:10.1245/s10434-026-19931-4)
Supplement: Supplementary file 1 [file 10434_2026_19931_MOESM1_ESM.docx]

**Supplementary Materials - Index**

| **Supplementary Methods** |  |
| --- | --- |
| Supplementary Methods | *pag. 3* |
| **Supplementary Results** |  |
| Supplementary Materials 1 | *pag. 4* |
| **Supplementary Figures and Tables** |  |
| **Figure S1.** Receiver Operating Characteristic (ROC) curves of the radiomics model classifying failure versus success of active surveillance (AS) based on imaging features from T1-weighted sequences (including fat saturation), T2-weighted sequences (including fat saturation), or both. | *pag. 5* |
| **Figure S2.** Calibration plot of the multi-sequence radiomics model for all centers combined. The observed event rates are plotted against the mean predicted probabilities, with error bars indicating 95% confidence intervals. The dashed line represents perfect calibration. | *pag. 6* |
| **Figure S3.** Univariate analysis of imaging features associated with failure of active surveillance (AS) using the Mann–Whitney U test | *pag. 7* |
| **Table S1.** Several properties of the acquisition protocols of the MRI sequences of patients in all cohorts. | *pag. 8* |
| **Table S2.** Performance metrics of the radiomics models, including clinical, single and multi-sequence models, predicting progressive disease (PD) for the three separate cohorts (external validation). | *pag. 9* |
| **Table S3.** Overview of the 564 features used in this study. | *pag. 10* |
| **References** | *pag. 12* |
|  |  |

**Supplementary Methods**

This supplemental material is similar to the works of Timbergen et al.^1^ and Spaanderman et al.^2^ who used the same radiomics software^3, 4^.

A total of 564 radiomics features were included in this study. Feature extraction was performed using the default settings for MRI scans provided by the Workflow for Optimal Radiomics Classification (WORC) toolbox (version 3·7·0)^3, 4^, which internally incorporates the PREDICT (version 3·2·0) and PyRadiomics (version 3·0·1)^5^ toolboxes. A complete list of all extracted features is provided in **Supplemental Table S3**. For a detailed description of the mathematical definitions of these features, we refer to Zwanenburg et al. (2020)^6^. Additional information can be found in the documentation of the respective toolboxes, primarily in the WORC documentation^4^. The code used for feature extraction is publicly available as open-source^4^.

The features can be categorized into several groups. Thirteen intensity-based features were computed from the histogram of voxel intensities within the Regions of Interest (ROIs), i.e., the tumors. These include first-order statistics such as mean, standard deviation, and kurtosis, which characterize the intensity distribution within the lesion. Thirty-five shape features, derived solely from the ROI geometry (i.e., without using image intensity), capture morphological aspects such as volume, compactness, and circular variance. Nine orientation features were also included, describing the spatial orientation of the ROI independently of the image. Finally, 507 texture features were extracted to capture more complex image patterns. These included features from Gabor filters (156), Laplacian of Gaussian filters (39), vesselness filters targeting tubular structures (39)^7^, the Gray Level Co-occurrence Matrix (144)^6^, the Gray Level Size Zone Matrix (16)^6^, the Gray Level Run Length Matrix (16)^6^, the Gray Level Dependence Matrix (14), the Neighborhood Grey Tone Difference Matrix (5)^6^, Local Binary Patterns (39)^8^, and local phase filters (39)^9, 10^. These texture features are designed to quantify characteristics such as heterogeneity, presence of blob-like structures, and linear patterns within the lesion.

The dataset used was heterogeneous with respect to imaging protocols, which can influence feature values and limit the robustness of 3D features. To mitigate this, features were extracted per 2D axial slice and subsequently aggregated across slices, following WORC’s default approach^3^. The images were not resampled to avoid introducing interpolation artifacts. Given the variability in MRI scanner models, magnetic field strengths, and scan protocols, image contrast was highly variable and could influence feature extraction. To address this, each 3D MRI volume was normalized using z-scoring prior to feature extraction, which is also a default setting in WORC^3^. After extraction, several first-order statistics of the resulting feature distributions were calculated and used in the subsequent machine learning analysis.

**Supplementary Results**

**Supplementary Materials 1**

**Robustness analysis for radiomics-models**

The robustness of the radiomics model was evaluated in external validation by stratifying performance across demographic and imaging-related subgroups. Stratification factors included age (≤38 versus >38 years), sex (female versus male), tumor location (abdominal wall versus extremities versus chest wall versus other), MRI field strength (≤1.5 T versus >1.5 T), MRI vendor (GE, Siemens, Philips), fat saturation (FS versus non-FS), and imaging availability (imputation required for one sequence versus not required).

Radiomics model performance showed moderate robustness across most patient and imaging subgroups, although some variation was observed. Stratification by age revealed comparable performance in younger (≤38 years, AUC = 0.69 [0.58, 0.80]) and older patients (>38 years, AUC = 0.67 [0.54, 0.80]). A trend toward higher performance was noted in female patients (AUC = 0.71 [0.62, 0.81]) compared to males (AUC = 0.58 [0.40, 0.76]), though confidence intervals overlapped. Performance varied by tumor location, with higher AUCs for extremities (0.77 [0.60, 0.91]) and abdominal wall (0.72 [0.59, 0.84]), and lower values for chest wall (0.61 [0.34, 0.86]) and other locations (0.41 [0.15, 0.70]). Higher performance was observed for MRI scanners with field strength >1.5 T (AUC = 0.83 [0.71, 0.93]) compared to ≤1.5 T (0.65 [0.56, 0.75]), suggesting possible influence of scanner quality. Among MRI vendors, Siemens yielded the highest performance (AUC = 0.77 [0.66, 0.87]), followed by Philips (0.68 [0.54, 0.81]) and GE (0.62 [0.37, 0.85]). Fat-saturated and non-fat-saturated sequences showed similar performance (AUC = 0.71 [0.59, 0.82] versus 0.66 [0.54, 0.78]). Some reduction in performance was observed in patients requiring imputation of missing features (AUC = 0.59 [0.43, 0.73]) compared to those with complete data (0.73 [0.63, 0.82]). Although most subgroups showed overlapping confidence intervals, the observed trends suggest that scanner characteristics, vendor differences, and imputation may impact model performance to some extent

**Supplementary Figures and Tables**

**Figure S1.** Receiver Operating Characteristic (ROC) curves of the radiomics model classifying failure versus success of active surveillance (AS) based on imaging features from T1-weighted sequences (including fat saturation), T2-weighted sequences (including fat saturation), or both.

LOCO = leave-one-country-out.

Model performance is reported as the area under the ROC curve (AUC) with corresponding 95% confidence intervals (CI). For external leave-one-country-out (LOCO) validation across different cohorts, CIs were calculated using 1000x bootstrapping. For the internal cross-validation experiment, where all cohort data were combined, CIs were derived from 100x repeated cross-validation test folds, ensuring the model had no prior exposure to the images used for prediction. Note, sample sizes differ slightly between T1-, T2-, and multi-sequence (T1+T2) models due to missing sequences in some patients. For the multi-sequence model, missing features were imputed, allowing the inclusion of all patients in the analysis.

**Figure S2.** Calibration plot of the multi-sequence radiomics model for all centers combined. The observed event rates are plotted against the mean predicted probabilities, with error bars indicating 95% confidence intervals. The dashed line represents perfect calibration.

**Figure S3.** Univariate analysis of imaging features associated with failure of active surveillance (AS) using the Mann–Whitney U test.

Each feature is shown for all three centers individually. Colors indicate the feature group; features

that reached statistical significance (below the first red dotted line) in at least one center are colored, while non-significant features are greyed out. Bonferroni-adjusted significance is indicated by the top red dotted line. A) Features derived from T1-weighted imaging (including fat-saturated sequences). B) Features derived from T2-weighted imaging (including fat-saturated sequences).

**Table S1.** Several properties of the acquisition protocols of the MRI sequences of patients in all cohorts.

|  | **The Netherlands**  **N = 72** | **Italy**  **N = 62** | **Canada**    **N = 66** | **P-value** |
| --- | --- | --- | --- | --- |
| **Magnetic field strength** |  |  |  | **<0.01** |
| **1T** |  | 9 (15) |  |  |
| **1.5T** | 60 (83) | 51 (82) | 31 (47) |  |
| **3T** | 12 (17) | 2 (3) | 35 (53) |  |
| **Manufacturer** |  |  |  | **<0.01** |
| **Siemens** | 21 (29) | 10 (16) | 59 (89) |  |
| **GE** | 11 (15) | 17 (27) | 7 (11) |  |
| **Philips** | 38 (53) | 35 (57) | - |  |
| **Unknown** | 2 (3) | - | - |  |
| **Setting T1 (Unit)** |  |  |  |  |
| **Slice Thickness (mm)^*^** | 4.1 ± 1.4 | 4.8 ± 1.3 | 3.8 ± 1.4 | **<0.01** |
| **Repetition time (ms)^*^** | 550 ± 647 | 472 ± 304 | 196 ± 441 | **<0.01** |
| **Echo time (ms)^*^** | 9.2 ± 6.0 | 10.5 ± 5.4 | 4.5 ± 5.9 | **<0.01** |
| **Setting T2 (Unit)** |  |  |  |  |
| **Slice Thickness (mm)^*^** | 4.5 ± 1.1 | 5.0 ± 1.3 | 5.1 ± 1.1 | **<0.01** |
| **Repetition time (ms)^*^** | 3926 ± 1837 | 3531 ± 2782 | 2373 ± 1871 | **<0.01** |
| **Echo time (ms)^*^** | 81.3 ± 23.7 | 92.2 ± 23.2 | 80.7 ± 13.5 | **<0.01** |
| **Available MRI sequences** |  |  |  | **<0.01** |
| **T1** | 47 (65) | 30 (48) | 41 (62) |  |
| **T1-FS** | 32 (44) | 8 (13) | 52 (79) |  |
| **T2** | 22 (31) | 45 (73) | 27 (41) |  |
| **T2-FS** | 31 (43) | 14 (23) | 53 (80) |  |
| **Segmented sequence** |  |  |  | **<0.01** |
| **T1** | 27 (38) | 10 (16) | 11 (17) |  |
| **T1-FS** | 10 (14) | - | 13 (20) |  |
| **T2** | 10 (14) | 39 (63) | 21 (32) |  |
| **T2-FS** | 7 (10) | 11 (18) | 16 (24) |  |

Values are n (%) unless otherwise indicated. P-values <0.05 are in bold.

* values are mean ± standard deviation.

**Table S2.** Performance metrics of the radiomics models, including clinical, single and multi-sequence models, predicting progressive disease (PD) for the three separate cohorts (external validation).

| **Cohort** | **AUC** | **BCA** | **Sensitivity** | **Specificity** | **P-value^†^** |
| --- | --- | --- | --- | --- | --- |
| **Progressive disease (PD)** | | | | | |
| *Cohort 1: The Netherlands* | | | | | |
| T1 and T2 imaging model | 0.51 [0.35, 0.66] | 0.50 [0.42, 0.58] | 0.12 [0.00, 0.24] | 0.88 [0.79, 0.98] |  |
| Clinical model | 0.53 [0.39, 0.68] | 0.54 [0.45, 0.63] | 0.19 [0.04, 0.34] | 0.88 [0.79, 0.98] | 0.86 |
| Imaging and clinical model | 0.47 [0.31, 0.63] | 0.53 [0.43, 0.62] | 0.19 [0.04, 0.35] | 0.86 [0.76, 0.96] | 0.91 |
| T1 imaging model^*^ | 0.43 [0.28, 0.59] | 0.54 [0.44, 0.64] | 0.21 [0.04, 0.37] | 0.87 [0.77, 0.98] |  |
| T2 imaging model^*^ | 0.65 [0.48, 0.81] | 0.55 [0.42, 0.68] | 0.29 [0.07, 0.51] | 0.81 [0.66, 0.95] |  |
| *Cohort 2: Italy* | | | | | |
| T1 and T2 imaging model | 0.52 [0.36, 0.67] | 0.58 [0.47, 0.69] | 0.32 [0.14, 0.50] | 0.84 [0.72, 0.96] |  |
| Clinical model | 0.64 [0.50, 0.79] | 0.54 [0.45, 0.62] | 0.16 [0.01, 0.31] | 0.92 [0.83, 1.01] | 0.12 |
| Imaging and clinical model | 0.53 [0.38, 0.68] | 0.57 [0.45, 0.69] | 0.36 [0.17, 0.55] | 0.78 [0.65, 0.92] | 0.59 |
| T1 imaging model^*^ | 0.48 [0.29, 0.66] | 0.50 [0.37, 0.63] | 0.20 [0.02, 0.38] | 0.80 [0.62, 0.98] |  |
| T2 imaging model^*^ | 0.64 [0.49, 0.79] | 0.63 [0.52, 0.74] | 0.38 [0.18, 0.57] | 0.89 [0.79, 0.99] |  |
| *Cohort 3:* *Canada* | | | | | |
| T1 and T2 imaging model | 0.65 [0.51, 0.80] | 0.59 [0.49, 0.70] | 0.27 [0.08, 0.46] | 0.91 [0.82, 1.00] |  |
| Clinical model^§^ | 0.60 [0.46, 0.75] | 0.49 [0.40, 0.58] | 0.14 [0.00, 0.28] | 0.84 [0.73, 0.95] | 0.61 |
| Imaging and clinical model^§^ | 0.67 [0.53, 0.82] | 0.60 [0.50, 0.70] | 0.27 [0.08, 0.46] | 0.93 [0.86, 1.00] | 0.68 |
| T1 imaging model^*^ | 0.67 [0.52, 0.83] | 0.62 [0.53, 0.72] | 0.25 [0.06, 0.44] | 1.00^‡^ |  |
| T2 imaging model^*^ | 0.67 [0.52, 0.83] | 0.62 [0.53, 0.72] | 0.25 [0.06, 0.44] | 1.00^‡^ |  |

AUC = area under the ROC curve, BCA = balanced classification accuracy.

Data are mean [95% confidence intervals] over bootstrap resampling iterations. The clinical model contained age, sex and tumor location features.

^*^Sample sizes vary slightly between T1-, T2-, and multi-sequence (T1+T2) models due to missing sequences. Missing features were imputed for the multi-sequence model, enabling inclusion of all patients. These differences prevented DeLong test comparisons.

^†^The reported P-values represent DeLong’s test comparing each model against the T1+T2 imaging model.

^‡^Confidence interval could not be calculated.

^§^Clinical model without CTNBB1 mutation status.

| Histogram  (13 features) | LoG  (13*3=39 features) | | Vessel  (12*3=39 features) | GLCM (MS)  (6*3*4*2=144 features) | | Gabor  (13*4*3=156 features) | NGTDM  (5 features) | LBP  (13*3=39 features) |
| --- | --- | --- | --- | --- | --- | --- | --- | --- |
| min  max  mean  median  std  skewness  kurtosis  peak  peak position  range  energy  quartile range  entropy | min  max  mean  median  std  skewness  kurtosis  peak  peak position  range  energy  quartile  entropy | | min  max  mean  median  std  skewness  kurtosis  peak  peak position  range  energy  quartile  entropy | contrast (normal, MS mean + std)  dissimilarity (normal, MS mean + std)  homogeneity(normal, MS mean + std)  angular second moment (ASM) (normal, MS mean + std)  energy (normal, MS mean + std)  correlation (normal, MS mean + std) | | min  max  mean  median  std  skewness  kurtosis  peak  peak position  range  energy  quartile range  entropy | busyness  coarseness  complexity  contrast  strength | min  max  mean  median  std  skewness  kurtosis  peak  peak position  range  energy  quartile range  entropy |
| GLSZM  (16 features) | | **GLRM**  **(16 features)** | | | **GLDM**  **(14 features)** | **Shape**  **(35 features)** | **Orientation**  **(9 features)** | **Local phase**  **(13*3=39 features)** |
| Gray Level Non Uniformity  Gray Level Non Uniformity Normalized  Gray Level Variance  High Gray Level Zone Emphasis  Large Area Emphasis  Large Area High Gray Level Emphasis  Large Area Low Gray Level Emphasis  Low Gray Level Zone Emphasis  SizeZoneNonUniformity  SizeZoneNonUniformityNormalized  SmallAreaEmphasis  SmallAreaHighGrayLevelEmphasis  SmallAreaLowGrayLevelEmphasis  ZoneEntropy  ZonePercentage  ZoneVariance | | Gray Level Non Uniformity  Gray Level Non Uniformity Normalized  Gray Level Variance  High Gray Level Run Emphasis  Long Run Emphasis  Long Run High Gray Level Emphasis  Long Run Low Gray Level Emphasis  Low Gray Level Run Emphasis  RunEntropy  RunLengthNonUniformity  RunLengthNonUniformityNormalized  RunPercentage  RunVariance  ShortRunEmphasis  ShortRunHighGrayLevelEmphasis  ShortRunLowGrayLevelEmphasis | | | Dependence Entropy  Dependence Non-Uniformity  Dependence Non-Uniformity Normalized  Dependence Variance  Gray Level Non-Uniformity  Gray Level Variance  High Gray Level Emphasis  Large Dependence Emphasis  Large Dependence High Gray Level Emphasis  Large Dependence Low Gray Level Emphasis  Low Gray Level Emphasis  Small Dependence Emphasis  Small Dependence High Gray Level Emphasis  Small Dependence Low Gray Level Emphasis | compactness (mean + std)  radial distance (mean + std)  roughness (mean + std)  convexity (mean + std)  circular variance (mean + std)  principal axes ratio (mean + std)  elliptic variance (mean + std)  solidity (mean + std)  area (mean, std, min + max  volume (total, mesh, volume)  elongation  flatness  least axis length  major axis length  minor axis length  maximum diameter 3D  maximum diameter 2D (rows, columns, slices)  sphericity  surface area  surface volume ratio | theta_x  theta_y  theta_z  COM index x  COM index y  COM index z  COM x  COM y  COM z | min  max  mean  median  std  skewness  kurtosis  peak  peak position  range  energy  quartile  entropy |

**Table S3.** Overview of the 564 features used in this study. GLCM features were calculated in four different directions (0, 45, 90, 135 degrees) using 16 gray levels and pixel distances of 1 and 3. LBP features were calculated using the following three parameter combinations: 1 pixel radius and 8 neighbors, 2 pixel radius and 12 neighbors, and 3 pixel radius and 16 neighbors. Gabor features were calculated using three different frequencies (0.05, 0.2, 0.5) and four different angles (0, 45, 90, 135 degrees). LoG features were calculated using three different widths of the Gaussian (1, 5 and 10 pixels). Vessel features were calculated using the full mask, the edge, and the inner region. Local phase features were calculated on the monogenic phase, phase congruency and phase symmetry.

COM = center of mass; GLCM = gray level co-occurrence matrix; MS = multi slice; NGTDM = neighborhood gray tone difference matrix; GLSZM = gray level size zone matrix; GLRLM = gray level run length matrix; LBP = local binary patterns; LoG = Laplacian of Gaussian; std = standard deviation.

**References**

1.	Timbergen MJM, Starmans MPA, Padmos GA, Grünhagen DJ, van Leenders G, Hanff DF, Verhoef C, Niessen WJ, Sleijfer S, Klein S, Visser JJ. Differential diagnosis and mutation stratification of desmoid-type fibromatosis on MRI using radiomics. *Eur J Radiol* 2020;**131**: 109266.

2.	Spaanderman DJ, Hakkesteegt SN, Hanff DF, Schut ARW, Schiphouwer LM, Vos M, Messiou C, Doran SJ, Jones RL, Hayes AJ, Nardo L, Abdelhafez YG, Moawad AW, Elsayes KM, Lee S, Link TM, Niessen WJ, van Leenders G, Visser JJ, Klein S, Grünhagen DJ, Verhoef C, Starmans MPA. Multi-center external validation of an automated method segmenting and differentiating atypical lipomatous tumors from lipomas using radiomics and deep-learning on MRI. *EClinicalMedicine* 2024;**76**: 102802.

3.	Starmans MPA, Voort SRvd, Phil T, Timbergen MJM, Vos M, Padmos GA, Kessels W, Hanff DF, Grunhagen DJ, Verhoef C, Sleijfer S, Bent MJvd, Smits M, Dwarkasing RS, Els C, Fiduzi FIF, Leenders GJLHv, Blažević A, Hofland J, Brabander T, Gils RAHv, Franssen GJH, Feelders RA, Herder WWd, Buisman FE, Francois E.J.A. Willemssen B, Koerkamp G, Angus L, Veldt AAMvd, Rajicic A, Odink AE, Deen MJ, Castillo JM, Veenland JF, Schoots IG, Renckens M, Doukas M, Man RAd, Ijzermans JNM, Miclea RL, Vermeulen PB, Bron E, Thomeer MGJ, Visser JJ, Niessen WJ, Klein S, Koerkamp BG. An automated machine learning framework to optimize radiomics model construction validated on twelve clinical applications. *arXiv* 2021.

4.	Starmans MPA, Voort Svd, Phil T, Klein S. Workflow for Optimal Radiomics Classification (WORC) Software. 2018.

5.	van Griethuysen JJM, Fedorov A, Parmar C, Hosny A, Aucoin N, Narayan V, Beets-Tan RGH, Fillion-Robin JC, Pieper S, Aerts H. Computational Radiomics System to Decode the Radiographic Phenotype. *Cancer Res* 2017;**77**(21): e104-e107.

6.	Zwanenburg A, Vallières M, Abdalah MA, Aerts H, Andrearczyk V, Apte A, Ashrafinia S, Bakas S, Beukinga RJ, Boellaard R, Bogowicz M, Boldrini L, Buvat I, Cook GJR, Davatzikos C, Depeursinge A, Desseroit MC, Dinapoli N, Dinh CV, Echegaray S, El Naqa I, Fedorov AY, Gatta R, Gillies RJ, Goh V, Götz M, Guckenberger M, Ha SM, Hatt M, Isensee F, Lambin P, Leger S, Leijenaar RTH, Lenkowicz J, Lippert F, Losnegård A, Maier-Hein KH, Morin O, Müller H, Napel S, Nioche C, Orlhac F, Pati S, Pfaehler EAG, Rahmim A, Rao AUK, Scherer J, Siddique MM, Sijtsema NM, Socarras Fernandez J, Spezi E, Steenbakkers R, Tanadini-Lang S, Thorwarth D, Troost EGC, Upadhaya T, Valentini V, van Dijk LV, van Griethuysen J, van Velden FHP, Whybra P, Richter C, Löck S. The Image Biomarker Standardization Initiative: Standardized Quantitative Radiomics for High-Throughput Image-based Phenotyping. *Radiology* 2020;**295**(2): 328-338.

7.	Frangi AF, Niessen WJ, Vincken KL, Viergever MA. Multiscale vessel enhancement filtering. In; 1998; Berlin, Heidelberg: Springer Berlin Heidelberg; 1998. p. 130-137.

8.	Ojala T, Pietikainen M, Maenpaa T. Multiresolution gray-scale and rotation invariant texture classification with local binary patterns. *IEEE Transactions on Pattern Analysis and Machine Intelligence* 2002;**24**(7): 971-987.

9.	Kovesi P. *Phase Congruency Detects Corners and Edges*, 2003.

10.	Kovesi P. Symmetry and asymmetry from local phase. In: In Tenth Australasian Joint Conference on Artificial Intelligence: Citeseer; 1997. p. 2-4.
